# A qualitative exploration of the therapeutic characteristics of the art of therapy: Perspectives on Ayres Sensory Integration

**DOI:** 10.1371/journal.pone.0322433

**Published:** 2025-05-08

**Authors:** Beverley Margaret Williams, Jean Daly-Lynn

**Affiliations:** School of Health Sciences, Ulster University, Belfast, Northern Ireland; Public Library of Science, UNITED KINGDOM OF GREAT BRITAIN AND NORTHERN IRELAND

## Abstract

Since Dr. Jean Ayres first ignited the spark that became Ayres Sensory Integration® others have followed, inspired by her legacy to continue her dedicated life’s work. This theoretical approach continues to evolve, yet its fundamental core principles remain true to Dr Ayres’s original ideal. Empowering practitioners to set the just right challenge, an inviting therapeutic relationship, and to entice engagement through play was conceptualized as the art of therapy. This qualitative phenomenological study explores the therapeutic characteristics of the art of therapy, emphasizing the just right challenge, the therapeutic relationship and the experience of flow from the occupational therapist’s viewpoint. Eight participants kept reflective journals and engaged in semi-structured interviews which were analyzed using reflective thematic analysis. Three overarching themes were developed; 1) “The Human Connection” exploring components of the therapeutic relationship; 2) “Setting the Scene” investigating how the just right challenge is set, including the preparation and external factors involved; 3) “Magic Happens! The Optimal Experience” exploring a sense of achievement, how flow feels and how it is learned. Participants shared their unique therapeutic observations and provided clear insight into their understanding of the art of therapy. The findings demonstrated that this concept is complex and multifaceted with learning enhanced through mentorship and hands-on experiences. This highlighted the significant implications for the education and ongoing mentoring of both practicing and new clinicians. This study provided novel insight into the field of occupational therapy using Ayres Sensory Integration® by developing a deeper understanding of therapists’ experiences of the art of therapy.

## Introduction

Sensory integration is explained as the process of detecting, registering, integrating and organizing the sensory information received from our bodies and the environment [1]. The process of interpreting information from the senses, such as sight, sound, touch, taste, smell, vestibular and proprioception, are critical to development and enables meaningful and purposeful participation in all aspects of life [2]. Impaired sensory integration impacts occupational performance including participation in activities, tasks and occupations, this, in turn, has an impact on behavior, self-esteem and emotional regulation [3–6].

Atypical sensory integration is recognized in children with Autistic Spectrum Disorder (ASD), Attention Deficit Hyperactivity Disorder (ADHD) and Developmental Coordination Disorder (DCD), as well as children with no formal diagnosis [7–9] and continues across the life span [10–12]. In a study from the United States of America (USA), atypical sensory patterns were found to be present in both children with typical development, and those with developmental conditions. The authors suggested this was likely to be reflective of the general population [13].

Ayres Sensory Integration® (ASI®) is an evidence-based practice for children with ASD [14–15]. Sensory integration therapy (SIT) is often the term used in the United Kingdom (UK), but this is referred to globally as Ayres Sensory Integration® (ASI®). The term ASI® will be used throughout this study. ASI® is a trademarked therapeutic practice well-established in occupational therapy (OT) [16–18]. Originally developed by Dr A. Jean Ayres in the 1950’s, this theory-based frame of reference hypothesizes that efficient integration of sensation is required for learning; that difficulty with processing and integrating sensation has an effect on function and behavior; and that sensory integration can be improved by providing sensorimotor opportunities that facilitate an adaptive response, and in turn, enhance function and behavior [19]. The last decade has seen an increase in the evidence of the effectiveness of ASI® such as improvements in performance and functional goals including play, social participation and self-care skills in children with ASD [14,20,21].

ASI® is typically carried out in a clinic-based setting using a range of suspended and floor-based equipment which provide opportunities for individually-tailored challenges with the aim of improving specific sensory systems that interfere with function and behavior [1,22]. It is child-led and draws on the intrinsic motivation to engage and experience a sense of achievement, in collaboration with the therapist. Activities are therapeutically facilitated within the context of play and exploration and are set at the just right level to promote an adaptive response. The challenge should be set just beyond the child’s capability to promote interest whilst not being so difficult that the child gives up [2]. The structuring of the therapeutic environment, to achieve the adaptive response, demands that the therapist is aware of the child’s motor capabilities, and has special understanding, ability and considerable skill [21]. Therapists’ need to be attuned to the child’s arousal state, mindful of the child’s sensory threshold and in tune with the child’s engagement level [23].

Stackhouse [24] surmised that the therapist can ‘scaffold’ activities through grading, moderating or structuring, to elicit the just right challenge and promote an adaptive response. Theory suggests that this pairing of skills promotes neuroplasticity, and is considered the basis for the art and science of ASI® treatment. When the just right challenge is achieved, the child switches on signifying that the activity is graded at the correct developmental level to allow maturation of the central nervous system [18,25]. Vygotsky in the 1920’s identified the gap between independent learning and what can be achieved with guidance and scaffolding, and named this concept the zone of proximal development. It was observed that individuals learn best when collaborating with more skilled persons, and that it is through these endeavors that they internalize new concepts, psychological tools and skills [26].

Flow is another characteristic of the therapeutic process, and is not exclusive to ASI® or OT but can be considered an aspect of human nature. Csikszentmihalyi [27] described flow as a state of complete absorption in which those experiencing it have no regard for hunger, fatigue, or discomfort, whilst Gold & Ciorciari [28] compared flow to a spiritual experience. To be in flow, the level of difficulty of the task must perfectly match a person’s skill level with just manageable challenges [29], this is intrinsic to ASI®.

Bundy and Lane [30] note that:

“*Artful practitioners respond to complexity in what seems to be simple ways and without disrupting the flow. They make therapy look easy, even with the most difficult child and in the most difficult situation.*”

Ayres [2] described the art of therapy as being akin to psychotherapy, especially in relation to the attitude of the therapist. In either case, if the therapist chooses, they can feel with the person and empathize so closely that the experience can become one of their own.

*“Patient and therapist work together, and the togetherness is often felt quite* *consciously by the therapist.” Ayres (1972 P265).*

OT’s are facilitators of the art of therapy as they are experts in play analysis [31], activity grading and engagement through meaningful occupation [32]. They bring with them a host of personal and therapeutic skills; this is often termed the therapeutic use of self [33]. Bundy and Lane [30] consider that the therapeutic use of self, along with the ability to read the child’s cues, to set the just right challenge and to engage in play as equals, are vital components of the art of therapy.

There has been significant research in the field of ASI® [21], yet very little has been studied regarding the interaction between the child and therapist. Some early studies analyzed treatment sessions of OTs using an ASI® approach [34–35]. More recent, qualitative studies have explored both the perspectives on sensory-based interventions of parents of children with ASD [36] and the sensory experiences of autistic mothers [37]. Other qualitative studies have sought to gain insight from therapists in other fields, [38–39], however, no studies were found that focused on the experiences of OTs using an ASI® approach.

As the therapist’s use of self contributes to the success of an intervention, it is crucial to understand how that is internalized, particularly considering the level of effort and clinical reasoning involved in the process. In recent years, especially since the COVID 19 pandemic, ASI® education taught remotely, with synchronous videos, has become more evident, with the completion of clinical practice hours and senior mentorship being essential requirements for qualification. In this respect experiences can vary considerably, and although considered best practice, there is no current requirement for ongoing mentorship post certification. In these circumstances, the understanding of the art of practice can be lost.

The overarching aim of this study was to explore the experience of the art of therapy, from the therapist’s perspective, within which the therapeutic relationship, the just right challenge and flow seem to exist. Within that aim, we explored how flow feels in practice, and the variables perceived to either impede or facilitate success for the child. The characteristics of the art of therapy were explored through the lived experiences of the participating occupational therapists.

## Method

### Research design

An experiential, contextualistic, interpretivist approach underpinned this qualitative, phenomenological study to explore the lived experiences of therapists during ASI® therapy sessions. An interpretive stance was chosen as it provided a platform to gain in-depth understanding of the data [40]. Data was collected from participants’ reflective journals and from semi-structured interviews and was analyzed using reflective thematic analysis [41]. The study received approval from Nursing and Health Sciences Filter Committee at Ulster University. All participants provided written informed consent prior to the start of the study.

### Participants

A purposive sampling method was utilized to ensure that participants had sufficient experience and qualifications that would match the aims and objectives of the research. This would ensure that the data was trustworthy, thus improving the rigor of the study [42]. With permission from ASI-WISE, the internationally-accredited UK and Ireland provider of ASI® education, an expression of interest was sent to Telegram forums and special interest social media groups. This progressed to snowball sampling [43] as interested parties passed the inquiry to their professional contacts. Each interested party was emailed the study participant information sheet, a distress protocol, a reflective journal guide, an interview guide and consent form. The distress protocol was included as a means of responding to potential participant distress. Once consent forms were returned, participants were offered a one-week cooling-off period which gave time to ask questions. The recruitment activity took place between September 2022 and December 2022, and out of 36 therapists’ who expressed their interest 8 were recruited. There are no sample size guidelines for studies of this nature, but Marshall et al [44] recommend that the researcher allows time to devote sufficient attention to analyzing in-depth data. Furthermore, to gain information power rather than saturation, Malterud et al [45] suggest that fewer participants are needed, as long as the participants’ characteristics are very specific to a study’s aims, as is the case of this present study.

All participants were pediatric therapists in private practice as ASI® is not readily available from statutory services in the UK. The participants worked with a wide range of children and young people with varying degrees of sensory challenges from both mainstream and special schools. Their clients included those with learning difficulties, ASD, DCD, and childhood trauma. Inclusion criteria consisted of OTs who hold a qualification (post-graduate certificate, diploma, or the Collaborative for Leadership in Ayres Sensory Integration (CLASI) certification) in Ayres Sensory Integration®, had children on their caseload who had received ASI® within the last 6 months as part of their usual OT intervention, and who had internet access and a device to use for interview. Participants were excluded if they were not OTs by profession.

### Data collection

Participants were requested to complete a reflective journal for one month, prior to the interview taking place, to document their experiences, thoughts, and feelings, during ASI® sessions. Both Rebar [46] and Morrell-Scott [47] recognized that a diary provides unadulterated insight into phenomenological perspective as, being completed closer to the event, the information is likely to be more accurate and captures information that cannot be collected by interviews alone. This method is used successfully by researchers, albeit usually over a longer period of time [48–50]. A reflective journal protocol was provided to guide participants in their reflections (Table 1). This could be considered restrictive, however, Plummer [51] reported that solicited diaries ensure that relevant data is collected and that the writer remains focused on the research question.

**Table 1:** Reflective journal guide. The guidance given to participants to complete the reflective journals.

Was this a successful session? Or were there barriers that hindered success?
What went well? What didn’t go as expected? What could I do next time?
Were adaptive responses observed? From your observations, did the client experience that feeling of “flow”? How could you tell? Please give examples.
How does “flow” feel from your perspective? Does it feel comfortable and natural? Or does it feel onerous or stressful?
Was scaffolding required to ensure success? If so, did that work? What was the result?
Did you sense a therapeutic alliance with your client? How did that feel? Could that be observed?

All participants returned their journals electronically to the researcher prior to their interviews taking place. Semi-structured 1:1 interviews between the first author and the individual therapists’, and lasting between 30 and 60 minutes, were completed virtually. Semi-structured interviews have been successful in previous research seeking to understand a therapist’s perspective [52–53]. The reflective journals were utilized to supplement the conversation during the interview [54] and to form a picture of what the researcher was not able to observe directly. According to Duke [55], journals are more effective when used in conjunction with other research tools.

Interviews were video-recorded and transcribed using the Microsoft Teams transcription facility. Interviews were guided by the topic guide provided (Table 2) and by referring to the individual’s reflective journal. Video interviewing is a visual resource in addition to auditory information, as Ganguly [56] discusses, it allows the researcher to document important nonverbal cues, which would otherwise be missed.

**Table 2:** Interview guide The topic guide used in the semi-structured interviews.

How were you able to manage your reflective diary for the period specified?
Can you tell me about any available time that you had to reflect on your entries?
Tell me a little about your experience of using ASI within your practice and your usual client group?
Can you identify the ‘just right challenge’ and that feeling of ‘flow’ within a session as it’s happening?
When in that moment, could you give an example of how you might adapt an activity to facilitate an adaptive response?
This high clinical reasoning is complex and has been described as ‘dynamic assessment in motion’, could you describe how that feels to you?
What questions are you asking yourself when you are in that moment?
Much effort is placed on post graduate training in the field of SI, but what do you feel was the one factor that helped you to learn or understand that meaning of ‘flow’?
Not all sessions feel successful, how do you know when you’re not achieving flow- when you’re stuck in that process? Could you give an example?
What do you feel the most important element of preparation is to facilitate the feeling of flow and the therapeutic alliance?

Transcriptions were manually reviewed for anomalies following each interview and confidentiality of the participants was protected throughout the process by removing all identifiable information. To provide anonymity for data analysis, each participant was given a pseudonym at this stage. Throughout the data collection process, the researcher maintained a reflective journal.

### Data analysis

Reflective thematic analysis (RTA) developed and then further expanded by Braun and Clarke [57–58] was adopted as it offers an interpretative perspective. In addition, a hermeneutics of empathy stance was taken to help the researchers stay close to the original meaning provided by the participants whilst being aware of their own bias and assumptions [59].

The data collection and analysis were carried out by the first author who having firsthand experience of OT using ASI®, and a wealth of knowledge from their experience of the art of therapy, could be termed an ‘insider researcher’ [41]. Reflective thematic analysis was specifically chosen as it embraces subjectivity, which is not viewed as problematic but valued as an integral part of the analysis process [60–61] Furthermore, Trainor and Bundon [62] state that reflexivity provides transparency throughout the research process to share challenges, feelings, and realizations.

Rather than a rigid structure, RTA provides guidelines for data analysis [41] which includes familiarization, coding, generating, refining, and re-defining themes. Throughout the process, to ensure that no significant theme was missed, data analysis remained fluid as it evolved. This model allows for inductive exploration which is important to capture both semantic and latent meaning of the data.

Following the interviews, the first author listened back to each one and read the transcriptions twice to be fully immersed in the data and to become familiar with the content. From there, codes were identified and placed on an electronic table to capture individual concepts. Shared experiences and patterns of thought were clustered together manually, reviewed and edited several times. The authors discussed the patterns identified in the data and this discussion created depth to the analysis. As themes were developed, they were placed in an electronic table with the associated data to help the researchers in making sense of the data. Refinement was one of creative exploration which continued to evolve throughout the process as deeper and more meaningful themes emerged. During this process, the first author documented feelings and thoughts to aid consideration of their own influences and subjectivities. This formed part of the data analysis and points for discussion.

## Findings

Three main themes were developed during the thematic analysis process and are illustrated below (Table 3). For this study, the participants were not required to provide information on their client group. For ease of reading, the client is referred to as the child throughout the text. All quotes provided relate to participant pseudonyms and refer to data from interviews, unless otherwise stated.

**Table 3:** Themes. The themes and sub-themes that were developed through reflective thematic analysis.

Theme	Sub-theme
The Human Connection	Sensibility
	You Can Count On Me
	Common Ground
Setting The Scene	Be Prepared! Everything In Its Place
	Extraneous Circumstances
Magic Happens! The Optimal Experience	Mindfulness In Action
	Magic! Self-actualization
	The Artful Therapist

### Theme 1: The human connection

The theme *The Human Connection* explores the depth of the dyadic relationship between child and therapist. In this instance we explore the relationship during OT using ASI® intervention and specifically within the context of the art of therapy. The three sub-themes of ‘Sensibility’, ‘You Can Count On Me’ and ‘Common Ground’ discuss the therapist’s ability to make connections that support the therapeutic relationship as experienced by the participants. There was a common assumption that this strong therapeutic alliance developed trust and was at the core of all intervention, thus facilitating flow.

#### Sensibility.

The sub-theme ‘Sensibility’ describes the therapist’s emotional responsiveness to the child. It was evident that they instinctively remained close in both a physical and emotional sense. They were fully aware of, and immersed in, the child’s physical and emotional well-being, which allowed them to respond quickly and effectively to the many nuances that create bonds. Many of the participants talked about feeling fully connected to the child in every sense of the word, not only to provide a safe physical environment but also to create an emotional safe space.

*“And I suppose the principles of that kind of intensive interaction and that mirroring, and I suppose in general body language, if we mirror somebody’s body language, we feel more connected. I think, I don’t know that I do that deliberately necessarily, but I think it’s part of the reflections. I noticed it, so being in close proximity is the key, being close to them. Firstly, for safety but also just connectedness as well.”* **Chloe**

More than half the participants described this connection as a two-way process or a back-and-forth exchange of gestures and responses, both emotional and physical. Several participants described this as a dance between two people and most used words such as ‘wavy’, ‘fluid’, and ‘harmonious’.

*“I mean, the dancing one is kind of like I don’t know it’s when you dance in partnership, you don’t always have to do a ballroom dance or whatever. You could just dance with someone, it doesn’t have to be staged or anything, but it’s like a back-and-forth exchange.”* **Beth**

All participants described feelings of ease, calmness, comfort and safety within their journals, yet verbally this was difficult to describe:

*“It’s so difficult to find words for that. It’s kind of like we’re both going with the same plan, although there is no plan. It’s like when you walk with somebody and you’re walking at the same rhythm.”* **Lynne**

Half the participants agreed that connection did not always happen spontaneously and could be difficult to achieve. There were times when they needed to dig deeper and work harder to strengthen that bond. Several participants felt this was the case with non-verbal clients and those with oppositional tendencies, although not always, as one participant described:

*“It really made me think about how there’s some children, I mean, I don’t (click with). It’s not comfortable being this honest, but how some sessions I really look forward to (not others).”* **Katy**

#### You can count on me.

The sub-theme ‘You Can Count On Me’ gives insight into the foundational structure of the emotional bond between the therapist and child. As described by all participants, trust, safety and security were common building blocks on which therapeutic relationships were built. This connection led to a mutual understanding where the therapist provided a safe haven for play:

*“I was conscious of making a safe space for him to be, where minimal communication was accepted, where his consent was continually sought.”* **Mary** (extract from reflective journal)

Trust and safety facilitated the child’s willingness to engage, which led to an increased sense of purpose and self-esteem:

*“He had the most amazing play session. It was so free. He was doing things that I’ve never seen him do before and he was hanging upside down and doing all sorts of things.”* **Lynne**

Use of the child’s favorite characters and dreamscapes within imaginary play narratives was seen as essential by all participants. Not just to define motivators but, in addition, to facilitate trust and create the feeling of safety and acceptance which children found reassuring:

*“If your whole world is Thomas the Tank and you come into a new room with a new person, and then there’s this Thomas the Tank, that’s immediately fun and reassuring, so, I do like to know about the person’s interests and try to include that.”* **Katy**

Being seen as a safe person and engaging in the child’s narrative was important to facilitate that playful flow of engagement. This was observable through joyful interactions and repetitive play:

*“Laughing, and the fact that he was continuing, and he was controlling it, and he was comfortable, and he felt safe, and it was just flowing. It just flowed so well because it was so enjoyable, and it happened so naturally when he was responding and laughing and saying he wants to do it again.”* **Beth**

#### Common ground.

This sub-theme explores the process of collaboration through play and expands on the therapeutic relationship. Therapists inferred that they took equal responsibility for working together towards united goals, not necessarily therapeutic goals, but child-focused play goals. All participants expressed the belief that finding common ground to collaborate on a chosen activity through play was core to their sessions:

*“The child feels that collaboration because you’re paying attention to what is theirs. They’re motivated by their priority, what’s their preference, and so on, and you’ll then weave that into your targets and the goals you’ve got in the back of your mind for that child.”* **Jayne**

Another participant emphasized the need for mutuality:

*“Yeah, it’s that shared aim to achieve. I think it’s helpful and I think that’s where the flow is more natural. Whereas if it was my goal, and I was trying to drag him along for the ride, he ain’t gonna be in flow doing that.”* **Sarah**

Connecting through play was seen by many as experiential and heavily dependent on childhood environments, personality and intrinsic qualities:

*“I would say that (being playful) was a strength in my practice, but I think a lot of that comes from my childhood and my experiences there. I think you know that’s very much influenced how I can connect with children, and you know if you can join them at their level in their world, I think that’s a really good starting point to kind of achieve.”* **Chloe**

Yet the ability to be playful did not come naturally to all – a few described how, at times, they felt out of their comfort zone:

*“it’s almost like my gold standard creativity is to take this child to another world of play, but I find play so hard, so my ideation practice is not brilliant. So, for me I find that really unnatural to do. For me to get into flow is really, really, tricky, but I’m always really, mindful of that.”* **Sarah**

More experienced therapists considered collaboration through play to be a completely natural phenomenon, not just part of the ASI® approach but a fundamental part of their OT persona:

*“But yeah, I think as OT’s we have a fabulous variety of tools in our toolbox that we pull out inside sessions, and I guess that’s that therapeutic use of self, and what we have in the back pocket kind of thing.”* ***Anne***

This deep collaboration, play and bonding experience was considered of high importance and rewarding for many participants. Connection was the metaphorical carrot that drew the therapists to OT in the first instance and then to using ASI®.

*“Yeah, it’s really rewarding and just like any relationship, if you’re both connected, if you’ve both got that shared enjoyment, it is a very rewarding process which again I think is why I love that part of my job so much. So yeah, I love it. It’s great.”* **Chloe**

### Theme 2: Setting the scene

The theme ‘Setting The Scene’ examines the therapist’s view of facilitating success for the child by grading and scaffolding activities to set the just right challenge. The sub-theme ‘Be Prepared! Everything In Its Place’ describes the influential factors that lead to success whilst the sub-theme ‘Extraneous Circumstances’ explores influences that could be considered barriers to success.

#### Be prepared! Everything in its place.

The sub-theme ‘Be Prepared! Everything In Its Place’ considers essential preparation for the child’s success through the just right challenge. All participants agreed that a thorough assessment was vital, not only to get a good understanding of the child’s sensory and functional challenges but also to understand what drives and motivates them.

“K*nowing the child really well, having done that comprehensive assessment, knowing what’s going on in their neurology and knowing their interests and what will grab them and keep them on board. Then you get that willingness to challenge, and you get the adaptive responses.”* **Jayne**

Preparation was conceptualized as being interlaced with connection and was seen as influential in building the therapeutic relationship:

“*So, I think because I insist on a thorough assessment, that means I get plenty of opportunities to meet them and work with them before we get to treatment, which means we already know each other and we’ve already got that rapport, and I already know more about their regulation and things, so when they come in for sessions, we’re already that step further forward.”* ***Sarah***

It was clear that both the assessment and therapeutic relationship were required to set the stage for the just right challenge and subsequently for flow to occur. The importance of scaffolding was recognized by all, this included subtly moving or stabilizing equipment, using a well-placed hand for physical support, and grading activities to the just right level:

*“You couldn’t do this therapy without scaffolding as one of the ingredients. It’s like play, you also need this as its part of the recipe! The scaffolding process supports and helps to organize the behavior within the session.”* **Beth**

#### Extraneous circumstances.

The sub-theme ‘Extraneous Circumstances’ describes how, despite best efforts, things don’t always go as planned. Most participants described several variables that were outside of their control including illness, tiredness, boredom or changes in routines. All impacted the child’s ability to maintain an optimum state of regulation and led the therapist to work harder to manage that session successfully.

On those occasions, some therapists struggled with the in-moment clinical reasoning and sometimes lost focus. They spoke of how, when a child fails, or is not fully engaged, flow was paused or stopped altogether, and this can affect the therapeutic relationship, albeit temporarily:

*“The opposite of that (success), is when I’ve got a child and I’m actually, I don’t want to say panicking, but I’m almost thinking, what we’re gonna do next? They’ve finished that and they’re almost there., almost stood there waiting for something, or the parents looking like I should be doing something. You know now and again there’s that bored feeling, not, that anyone’s ever said like I’m bored now, but sometimes it’s almost that feeling of I’m bored now and I’m thinking right? What’s next? Where is this?”* **Katy**

Several therapists recognized the need to allow time for the child to regulate before placing additional demands on them. The most common variable was the impact of caregivers in attendance, they often struggled to remain on the sideline, instead acting on their intrinsic need to parent, as shown in one participant’s reflective journal:

*“There were still barriers and Mum often wants to jump in and help child when child struggles and tell child that their ideas won’t work. I think she feels frustrated with the process. I have since spoken to her and she denies frustration at me but feels frustration towards her child because she feels he should know better.”* **Lynne**

In contrast, all participants reflected that caregivers can be a positive catalyst to the child’s success. Observation allows them to adopt therapeutic strategies which support the child outside of sessions. One therapist addressed this as follows:

*“Parents are present throughout my sessions as I think this helps build a relationship with them, which is also key to successful psychoeducation and trust building and also I’m demoing to them how to play with their kids which is another key deficit I often see.”* **Mary**

All participants reported that they provided goal-centered education to caregivers during or following sessions but not specifically education on the core principles of ASI®.

Several participants reported in their reflective journals that, when all was going well and the child was having fun, this can look and feel too easy. They felt this could be misjudged and that the therapeutic value of the intervention could be questioned. This momentary self-analysis sometimes interfered with the flow of the session, especially when cost was a factor. Being in private practice, some participants felt the pressure to ‘act’ or to look busy in case their value was questioned. One participant described it as follows:

*“it’s on your mind that these parents have paid you for this session and they’re expecting an hour of activity with their child and their child is in a really chilled place and he’s actually really happy just lying on the bean bags for a while.”* **Sarah**

### Theme 3: Magic happens! The optimal experience

The theme ‘Magic Happens! The Optimal Experience’ explores the therapist’s experience during moments of flow. The sub-themes ‘Mindfulness In Action’ and ‘Magic! Self-actualization’ refer to the concepts that were commonly discussed, which were considered the ultimate experience and outcome of therapy. The sub-theme ‘The Artful Therapist’ defines participants’ understanding of their learning process of flow and consequently the art of therapy.

#### Mindfulness in action.

The sub-theme ‘Mindfulness In Action’ refers to the feeling of total immersion in the moment and not consciously considering anything else at that time. Most participants expressed their minds as being fully occupied, with snippets of time when nothing else mattered outside of that moment. This allowed them to give their full attention to the present, as one participant described:

*“I don’t know. It’s just when you’re in that moment. You are very present in the moment with whatever is going on. If it’s an activity or if it’s a conversation, whatever it is, but you’re very invested in it in that moment.”* ***Lynne***

Half of the participants experienced time differently, commonly describing it as not existing outside of that moment. In her reflective journal, one participant described the following:

“*I see flow as being so absorbed in an activity that you temporarily lose awareness of time (lose track of time so time goes quickly, or time feels elongated through being occupied).”* **Mary**

This feeling was mutual and often experienced by the child. The sharing of occupied wonder was described as part of the therapeutic bond:

*“When experiencing flow – time flies so quickly, not thinking of anything else, feel very connected and tuned into the child – positive feeling.”* **Chloe** (extract from reflective journal)

When at the point of total absorption and full presence, several therapists described that there were no conscious decisions being made – their actions just felt spontaneous and natural.

*“This doesn’t feel like we’re practicing something. This doesn’t feel like I’m giving them an exercise they’re trying to fulfil. This just feels really natural.”* **Jayne**

Therapists’ talked about the high level of clinical reasoning that is expected during ASI® sessions, however, a few of the participants felt that this disappeared during those moments and any ‘thinking’ appeared to be on a subconscious level. This helped one participant recognize when flow was occurring:

*“So, if we’re running around, we’re playing a game and then suddenly I’m thinking, so you know that child needs to go on their tummy and I’m like, oh, grab these while you’re there and if that’s all just happening without me actually really consciously having to think, then to me that’s when they’re in flow.”* **Katy**

For others this was not the case and some participants struggled to think ahead in that moment:

*“(Flow feels) comfortable, although I can struggle with the forward planning or clinical reasoning whilst flow is ongoing!”* **Sarah**

#### Magic! Self-actualization.

The sub-theme ‘Magic! Self-actualization’ refers to the ultimate outcome during flow, that was sometimes described as magic by the participants. When both the therapist and child were described as being fully present in the moment and the just right challenge was set, only then could adaptive responses occur, or as some stated, the magic took place. All pre-planning led to this final moment:

*“I think the validation we get as therapists when it does happen is amazing. I think it does feel like magic. It’s like what just happened. It’s like a sort of disbelief that hits you.”* **Beth**

It was often difficult to verbalize the actual feeling or the moment in which the changes or adaptive responses occurred:

*“So, he really pushed himself to achieve and then we saw that it was almost like a flurry of things that happened after that. So, when he did it, it’s almost like it released something I don’t know, it’s really difficult to describe, it was a lovely session.”* **Lynne**

Many described the real sense of achievement and satisfaction for both therapist and child. At this point all movement was fluid, with no awkward pauses:

*“We don’t get too frustrated or stuck and she gives up, because it’s that sort of self-esteem, isn’t it? Of feeling like I can achieve it and it’s doable.”* ***Anne***

These moments were described as subconscious action for both parties, with the child’s success leading to increased confidence, wanting to repeat things again and again, and becoming intertwined with the environment:

*“He looked focused and somewhat ‘not in the room’. He had continually looked to mum for reassurance throughout the assessment (every 2 mins for 2 hrs.) but during the time on the equipment he didn’t look to mum nearly so much.”* **Mary** (extract from reflective journal)

Mutual respect was common and had lasting value for several participants. There was no hierarchy, no control of one person over the other – a feeling of mutual benefit:

*“I had this massive feeling of gratitude after his session for everything that he’s taught me (patience and time), and so, I think there’s something there about when you’re in a real kind of flow situation, it’s like a two-way relationship. It’s not that you’re just giving, you’re actually receiving from the child as well. I think that.”* **Katy**

#### The artful therapist.

This sub-theme gives voice to the therapists’ own experiences of learning flow, describing key factors that helped them in the transference of theory to practice. Almost all participants, regardless of the route they had taken for their ASI® qualification (e.g., virtual, in person, or a hybrid of both), felt that critical analysis of their work during education was important. At the same time, they were unanimous that the most influential factor in understanding flow was observing experienced therapists engaged in their craft. This was either achieved during in-person ASI® training or in their usual workplace:

*“It was really lovely to see how she (mentor) enabled the flow you know, because she had quite an imaginative back story if you like, to the session, that completely got this child engaged in it all.”* **Anne**

Most participants discussed the difficulty of learning the art of therapy, with the complexity of elements involved and the high level of clinical reasoning. Several felt that this cannot be taught, but is something that is learnt over time, and comes with experience confidence and maturity. When the in-person element was missing, the art of therapy was difficult to comprehend:

*“It almost feels like what they show you is like the top standard ideal world kind of thing, and you don’t learn about what to do when you got a kid who comes in who either can’t communicate, or you just can’t even regulate them. It’s so hard to even get them into a regulated state to even start your session.”* **Sarah**

Access to a mentor, who role-modelled the core attributes of ASI® and ignited a spark for further exploration, was commonly agreed as being influential during practice or training. This led to an ongoing commitment to learning, by all therapists. Ultimately, however, the most significant learning experience of all was centered around actual ‘seeing’ and ‘doing’.

*“Being in a whole room of really experienced therapists and them supporting me in things that I’d never done before…. Mm that was it.”* **Lynne**

## Discussion

This study explores the art of therapy and the main elements that are embedded within it, including the therapeutic relationship, the just right challenge and flow from the therapist’s perspective. Data shows that this process, tapping into unique qualities that shape capable therapists, is indeed an art. Being an insider researcher, the first author understands that her own values, assumptions and philosophy will have impacted the recording and interpretation of data in some way whilst also allowing exploration of this complex subject from an internal perspective.

The findings suggest that a strong bond between child and therapist emerged during the assessment process and continued throughout intervention. The theme ‘The Human Connection’, and specifically the sub-theme ‘Sensibility’, relate to the child and therapist being both physically and emotionally responsive to each other. This was the first step towards forming the relationship and is considered a two-way process. This mirroring of movements and body language is termed kinesthetic empathy and is a concept implemented in dance movement therapy (DMT). In her book on DMT, Fischman [63] explained this phenomenon as the ability to understand a person by attempting both to experience their inner life and to understand how they feel through non-verbal communication and body movements. The findings in this study are supported by Rova’s [6] practice-based interdisciplinary research project which compared a group of experienced dancers with a group of health care professionals. She found that kinesthetic responses were observed in both groups, regardless of prior experience, and that it exists in clinical practice.

Not all relationships flourish easily, and this can take time. Participants were honest in sharing that some work better than others. The sub-theme, ‘You Can Count on Me’ suggests that trust, safety and security are building blocks that strengthen the therapeutic relationship and current evidence supports this. In her book *The Intentional Relationship*, Taylor [6] breaks down these characteristics and conceptualizes that they make up the therapeutic use of self and are considered integral to Occupational Therapy. Crom et al [66] interviewed parents and children receiving physiotherapy intervention and found that parents valued therapeutic relationships more than skilled intervention.

The sub-theme ‘Common Ground’ highlights that providing a haven where children can be themselves, feeling valued and accepted, facilitates their willingness to engage. In a similar study by Lawton et al [6], semi-structured interviews were used to explore speech and language therapists’ perceptions and experiences of alliance. Similar qualities laid the groundwork for therapeutic relationships and were called recognizing personhood. The book *Clinician’s Guide to Implementing Ayres Sensory Integration®* [1] confirms that, to promote participation, the therapeutic relationship must be strong enough for the child to trust and attempt challenges where they might have previously experienced failure.

Collaboration, shared joy and playfulness were considered important factors in finding common ground which some therapists found challenging. Bundy and Lane [30] consider that, for therapy to be effective, both the therapist and child must be playing with the therapist seen as an adult playmate. The Royal College of Occupational Therapists (RCOT) [31] has since issued an occupational therapy and play practice guideline developed by Ward and Payne [68] which directs OTs to pay attention to play as an internal state of being and not just as an observable state of doing.

The theme ‘Setting The Scene’ and, importantly, the sub-theme ‘Be Prepared! Everything In Its Place’ suggests that participants endeavored to collaborate with the child and scaffold the just right challenge to facilitate success. This is consistent with the core components of ASI® and heavily supported in the literature [69–71]. The sub-theme ‘Extraneous circumstances’ describes caregivers as both barriers to and supporters of success. As to which one, this is mostly dependent on their understanding, and the level of information they received from the therapist. Previous research supports that parent/teacher education and participation is expected [72–73], yet often it is focused on goal-orientated coaching. Schaaf and Mailloux [1] developed a parent guidebook which Roan et al [74] adapted using knowledge translation strategies. This offers best practice strategies for educating parents of children with ASD who use an OT ASI® approach. If this parent guidebook is to be a common reference used in clinical practice, its content is likely to be adopted by other countries.

The theme ‘Magic Happens! The Optimal Experience’ and, in particular, the sub-theme ‘Mindfulness In Action’ suggests that the high level of in-moment clinical reasoning required during flow became an unconscious effort for some but proved challenging for others. The level of conscious thinking could relate to the ease of the therapeutic connection: when things were going well, therapists felt fully connected and completely immersed in the present moment. This is not unlike mindfulness as Germer [75] described this concept as being totally immersed in the task at hand. The findings in this study are supported by recent research by Smith et al [76] where mindfulness was found to be present in pediatric OT practices in Scandinavia. They concluded that mindfulness has the potential to support therapeutic relationships but advised that more research is needed.

The sub-theme ‘Magic! Self-Actualization’ discussed the therapist’s sense of achievement when intervention was successful. This was supported by the psychotherapist Kottler [77] in his book *On Being a Therapist*. The impact of client intervention on the therapist was explored and affirmed that the decision to become a therapist in whatever capacity was a commitment to self-growth and discovery.

Participants in this study described how the adaptive response occurring during moments of flow often felt like magic. Flow was studied by Csikszentmihalyi [27] who viewed it as the optimal experience. In their psychological study of flow in consciousness, Csikszentmihalyi and Csikszentmihalyi [78] discussed how flow has become a technical term used in the field of intrinsic motivation which supports its place within ASI® therapy. Yet flow is deeply rooted in psychology and relates to all human experience. The musician Slash, of Guns N Roses fame discussed his experience of flow, whilst playing guitar. He states in his autobiography [79] that with his hands occupied and his mind engaged he had truly found his inner peace. The findings in this study are supported by Solmon and Clouston [80] whose article elucidated that it is the role of the OT to use their therapeutic use of self to facilitate the optimal experience and to achieve the best outcome for their clients.

The sub-theme ‘The Artful therapist’ explored how participants learned to facilitate flow. It was evident that hands-on practice and observations of experienced mentors was crucial to their understanding of what it means to be an artful therapist. This is supported by a recent study by Rahman et al [81] that utilized questionnaires to assess how important and how confident 161 OTs in Malaysia using ASI® felt about the different components of intervention. Many of the participants were not yet certified in ASI® and learning had consisted of attending University courses only, with no mention of mentorship. They found that the majority of OTs considered the therapeutic relationship and setting of the just right challenge as very important, and they felt comfortable and confident in these areas. However, only 50% felt the same level of comfort and confidence in ensuring the child’s success, which could be an indication that the art of therapy has not been fully understood.

A recent study of mentorship for OTs in vocational rehabilitation by De Dios Pérez et al [82] supports that mentorship improves confidence and competence. Similarly, Schoen et al [69] studied the effectiveness of a specific mentorship program for OTs specializing in sensory integration and processing challenges. The results were positive which suggests that there is a need for more in-person, structured, mentored learning experiences.

## Implications for practice and future research

Throughout this study, practitioners have voiced their honest opinions by disclosing their concerns and sharing highlights of their intervention. Much of the data confirms what is already known regarding the art of therapy in the context of ASI®. Findings confirm the therapeutic relationship as multilayered and dependent on the therapeutic use of self. This is supported by current research [64,65,67] and is intrinsic to OT [65]. Yet the findings demonstrated how experienced therapists described some relationships as being difficult to cultivate. It is therefore suggested that, at entry level, more emphasis should be placed on assisting students to fully understand the building blocks of the therapeutic relationship, especially in relation to complex client groups.

The importance that participants placed on collaboration, setting the just right challenge, and flow within the art of therapy, showed their level of commitment and adherence to ASI® principles. It is crucial to develop these in caregivers to ensure better outcomes for their children. As discussed, clinicians in the USA have already developed a best practice guide on educating others [1,74]. It is suggested that clinicians in the UK consider doing the same. This would be a valuable asset, not only to assist therapists in supporting caregivers during intervention, but also to promote the fundamental principles of OT.

Findings indicate that participants experience feelings akin to mindfulness which emerging evidence supports as a therapeutic tool [76]. In recent years, mindfulness has emerged as an important concept for well-being and is used by many therapists and educators as well as in other areas of work [83–85]. It is suggested that further research is needed to explore its use in all fields of OT and with different client groups across the lifespan. Educators should consider its inclusion within curricula to help OT students using ASI® to fully understand the concept of the optimal experience and to improve practice and learning.

It was mentioned that much of OT and ASI® has moved to online learning which limits hand-on experiences for many. In recent research, students themselves have voiced their dissatisfaction with this approach [86–87] and further research is required to understand the impact that online learning has on skill acquisition and learning experience. Long et al [88] concluded that humans have a fundamental need for physical interactions and suggest that this cannot be replaced by online interaction; therefore, it is safe to assume that the move to virtual education has somewhat removed the concept of personhood and this is something which can limit the optimal experience of learning for many.

Whilst a virtual approach has its benefits, educators are strongly encouraged to reinstate hands-on practical workshops or modules. This would enable students with limited access to experienced workplace mentors, to fully understand how the art of therapy feels, as opposed to how it should look, to ensure the therapeutic elements are not lost. Schreiber et al [89] affirm that experiential learning provides critical exposure and opportunities that are fundamental to student development. In the present study, therapists who had learnt the art of therapy from skilled mentors, regarded them as integral to their learning process. This finding aligns with Husband [90] who highlighted that levering the skills and experience of mentors enhances professional learning. Collectively, these studies underscore the importance of incorporating both mentorship and hands-on learning opportunities into ongoing professional development for therapists, which could be effectively facilitated in small group settings.

This study gained novel insight into the therapist’s perspective of the therapeutic elements of OTs using ASI® and it is hoped that this study triggers experienced clinicians to reflect on their current practice. OTs working with children within the context of play, must develop a deep understanding of the art of therapy to ensure meaningful outcomes for their clients. It was important to reflect on current clinicians’ lived experiences to understand how that feels in practice.

### Limitations

The sample in this study was homogenous, consisting of eight female British occupational therapists all based in private practice. As such, findings may not be representative of either male clinicians, or those from different countries, cultures or services. In addition, there may be limitations to using self-reported diaries due to time and service pressure restrictions. This study focused on the use of ASI® with school-age children, however, the therapeutic components of this approach should be studied across the life span, and with varying client groups. This would broaden our understanding of the experiences of therapists, working outside of this specific client group, and would provide a comparison.

### Conclusion

This study provided novel insight into the therapist’s perspective on the art of therapy within the context of ASI®, emphasizing the therapeutic relationship, the just right challenge, and the experience of flow. The findings underscore that therapy is not merely a technical process but an embodied art that requires a deep, intuitive connection between therapist and child. Participants described how setting up the just right challenge, required both clinical reasoning and intuitive skill, which were best developed through hands-on experience and mentorship. The shift towards virtual education risks diminishing essential experiential learning opportunities that help therapists internalize the nuances of therapeutic presence, responsiveness, and flow. By capturing the lived experiences of occupational therapists using ASI®, this study contributes to a deeper understanding of how therapy feels in practice. It is hoped that these findings will encourage clinicians, educators, and professional bodies to reflect on current practices and consider how best to support the next generation of therapists in mastering the art of therapy.
